# A Human 2D Primary Organoid-Derived Epithelial Monolayer Model to Study Host-Pathogen Interaction in the Small Intestine

**DOI:** 10.3389/fcimb.2020.00272

**Published:** 2020-06-09

**Authors:** Thomas Roodsant, Marit Navis, Ikrame Aknouch, Ingrid B. Renes, Ruurd M. van Elburg, Dasja Pajkrt, Katja C. Wolthers, Constance Schultsz, Kees C. H. van der Ark, Adithya Sridhar, Vanesa Muncan

**Affiliations:** ^1^Department of Global Health-Amsterdam Institute for Global Health and Development, Amsterdam University Medical Center (UMC), University of Amsterdam, Amsterdam, Netherlands; ^2^Department of Medical Microbiology, Amsterdam University Medical Center (UMC), University of Amsterdam, Amsterdam, Netherlands; ^3^Tytgat Institute for Intestinal and Liver Research, Amsterdam Gastroenterology Endocrinology and Metabolism, Amsterdam University Medical Center (UMC), University of Amsterdam, Amsterdam, Netherlands; ^4^Viroclinics Xplore, Schaijk, Netherlands; ^5^Danone Nutricia Research, Utrecht, Netherlands; ^6^Department of Pediatrics, Amsterdam University Medical Center (UMC), Emma Children's Hospital, University of Amsterdam, Amsterdam, Netherlands; ^7^Department of Pediatric Infectious Diseases, Amsterdam University Medical Center (UMC), Emma Children's Hospital, University of Amsterdam, Amsterdam, Netherlands

**Keywords:** human organoids, small intestine, enteric pathogens, polarized epithelium, host-pathogen interactions, gut barrier

## Abstract

Gut organoids are stem cell derived 3D models of the intestinal epithelium that are useful for studying interactions between enteric pathogens and their host. While the organoid model has been used for both bacterial and viral infections, this is a closed system with the luminal side being inaccessible without microinjection or disruption of the organoid polarization. In order to overcome this and simplify their applicability for transepithelial studies, permeable membrane based monolayer approaches are needed. In this paper, we demonstrate a method for generating a monolayer model of the human fetal intestinal polarized epithelium that is fully characterized and validated. Proximal and distal small intestinal organoids were used to generate 2D monolayer cultures, which were characterized with respect to epithelial cell types, polarization, barrier function, and gene expression. In addition, viral replication and bacterial translocation after apical infection with enteric pathogens Enterovirus A71 and *Listeria monocytogenes* were evaluated, with subsequent monitoring of the pro-inflammatory host response. This human 2D fetal intestinal monolayer model will be a valuable tool to study host-pathogen interactions and potentially reduce the use of animals in research.

## Introduction

The intestinal epithelium consists of a single layer of polarized cells that separates the intestinal content from the rest of the body. Apart from facilitating absorption and digestion of food, it serves as an important barrier for warding off enteric pathogens such as viruses and bacteria (Odenwald and Turner, 2017; Allaire et al., 2018). This barrier function is primarily performed by highly polarized enterocytes with an apical brush border containing microvilli structures, which constitute the majority of cells in the intestinal epithelium (Crawley et al., 2014; Delacour et al., 2016). In addition to the enterocytes, Paneth and goblet cells counter luminal threats, by secretion of antimicrobial molecules such as defensins and mucins, respectively (Allaire et al., 2018). Junctional complexes (i.e., apical tight junctions and lateral adherens junctions) between these intestinal cells define paracellular permeability (Nusrat et al., 2000; Ivanov, 2012).

Understanding the interaction of pathogens with the intestinal epithelium and how they bypass the epithelial barrier is crucial for elucidating enteric diseases. Barrier dysfunction, for instance caused by microbial signals from pathogenic bacteria, can increase the susceptibility to infections and result in various intestinal and extra-intestinal diseases (Rahman et al., 2016; Ni et al., 2017; Manfredo Vieira et al., 2018; Rinninella et al., 2019). Traditionally, these host-pathogen interaction studies were performed *in vitro* using immortalized cancer cell lines which bear various mutations, resulting in aberrant proliferation and differentiation in comparison to *in vivo* intestinal epithelium (Sun et al., 2008; Maidji et al., 2017; von Martels et al., 2017; Calatayud et al., 2018). Another disadvantage of these models is the lack of diversity in specialized epithelial cell types, for example the Caco-2 cell line consists predominantly of enterocytes (Sun et al., 2008).

A major boost to modeling the intestinal epithelial barrier came with the advent of organoid models, which are stem cell derived 3D structures with a cell composition that is comparable to the *in vivo* situation (Sato et al., 2009, 2011; Nakamura, 2019). In recent years, organoids have emerged as a valuable model to study host-pathogen interactions at the intestinal epithelium *in vitro* (In et al., 2016; Mills and Estes, 2016; Yin and Zhou, 2018). For example it was shown that the human enteric pathogen *Escherichia coli* O157:H7 induces loss of epithelial integrity as early as 4 h after infection (Karve et al., 2017), and rotavirus preferentially infects differentiated cell types (Saxena et al., 2016). Since virus growth is limited in most transformed cell lines (Kitamoto et al., 1991), organoids offer advantages over existing cultures because they originate from primary cells. Moreover, the intrinsic cell heterogeneity of organoid cultures allows assessment of host cell type specific responses to infections in cultures from genetically diverse individuals.

However, studying translocation across the epithelium and, in particular, inoculation of only the luminal side is challenging in 3D structures. One way of accessing the luminal side is through microinjection (Hill et al., 2017; Williamson et al., 2018), but this method is technically challenging and reproducibility is still an issue, which limits its utility. Alternatively, the organoids can be mechanically disrupted to enable infection (Saxena et al., 2016), but with this approach the polarization is lost and there is both apical and basolateral contact of the epithelial cells with the pathogens. Therefore, several studies have proposed culturing intestinal organoids as a monolayer on permeable support (Moon et al., 2014; VanDussen et al., 2015; Kozuka et al., 2017; Noel et al., 2017; Horsley et al., 2018; van der Hee et al., 2018; In et al., 2019). In such a system, bacteria and viruses can be introduced to the culture medium directly on the apical (i.e., luminal) side of the epithelium. This monolayer culture can then be used to investigate host-pathogen interactions at the intestinal barrier. Such monolayer cultures have already been used to study pathogenic *E. coli* (ETEC and EPEC) (Noel et al., 2017), invasive cytomegalovirus, and enterovirus A-71 (EV-A71) (Maidji et al., 2017; Good et al., 2019).

With the increasing use of monolayer models, there is a need for development of standardized and reproducible protocols. In particular, biological variation between individuals is often unaccounted for with studies commonly featuring organoids obtained from a single biological sample. To address this, we established and validated in this study a primary human fetal intestinal epithelial model on Transwell^®^ cell culture inserts with organoids obtained from five different tissue specimens. To enable microbial translocation, inserts with membrane pore size of 3.0 μm were used, which is in contrast to mostly used membrane pore size of 0.4 μm for epithelial cells (Moon et al., 2014; VanDussen et al., 2015; Kozuka et al., 2017; Noel et al., 2017; Horsley et al., 2018; van der Hee et al., 2018; In et al., 2019). Proximal and distal fetal intestinal organoid monolayer cultures were characterized with respect to epithelial cell types, epithelial polarization, and epithelial function. The established model was next applied to study host-pathogen interactions at the intestinal barrier with a viral infection of EV-A71 and a bacterial infection and translocation of *Listeria monocytogenes*.

## Materials and Methods

### Ethics Statement

Human fetal intestinal tissue (gestational age 18–20 weeks) was obtained by the HIS Mouse Facility of the Amsterdam UMC from the Bloemenhove clinic (Heemstede, the Netherlands), with a written informed consent obtained from all donors for the use of the material for research purposes. These informed consents are kept together with the medical record of the donor by the clinic. Tissues were obtained with approval of the ethical committee of the Amsterdam UMC, together with approval of the experimental procedures by the HIS Mouse Facility (Amsterdam UMC). All methods were performed in accordance with the relevant guidelines and regulations, as stated in the Amsterdam UMC Research Code, in a certified laboratory (ISO15189 accreditation M304).

### Organoid Culture

To generate fetal organoids, fetal small intestine was macroscopically separated into proximal and distal using the location of the stomach and/or appendix. Subsequently, crypts were isolated as described previously (Sato et al., 2009). In short, human fetal small intestinal tissue was cut open longitudinally, cut into pieces of ~5 mm and extensively washed with cold PBS. Next, the tissue was incubated in 2 mM EDTA in PBS for 30 min at 4°C. Subsequently, the tissue was washed in PBS + 10% FCS and supernatant containing dissociated cells was collected and subsequently passed through a cell strainer (70 μm). Isolated crypts were resuspended in matrigel (Corning), dispensed in three 10 μL droplets per well in a 24 wells tissue culture plate and covered with 500 μL medium. Organoid cultures were maintained in Human IntestiCult™ Organoid Growth Medium (HIOGM, Stemcell™ Technologies) with 100 U-mg/mL penicillin-streptomycin (Gibco, Thermo Fischer Scientific) and incubated at 37°C, 5% CO_2_. Medium was refreshed every 2–3 days and organoids were passaged by mechanical disruption every 6–10 days as described previously (Sato et al., 2009), or by enzymatic dissociation as described for seeding onto cell culture inserts.

### Intestinal Organoid Monolayer Culture

Transwell^®^ cell culture inserts (6.5 mm, 3.0 μm pore size, VWR) were coated with 100 μL of 20 μg/mL collagen type I (rat tail, Ibidi) in 0.01% (v/v) acetic acid for 1 h at room temperature (RT), and washed with PBS before use. Human fetal organoids were expanded for one passage and subsequently collected at day 3. Single cell suspension was obtained by treatment with TryplE™ (Gibco, Thermo Fischer Scientific) for 10 min at 37°C. Cells were diluted to 1·10^6^ cells/mL and 100 μL of cell suspension per insert was seeded (=1·10^5^ cells per insert) unless stated otherwise.

Cells were cultured in 100 μL apical and 600 μL basolateral HIOGM for the first week, containing 10 μM Y-27632 (Stemcell™ Technologies) for the first 3 days. After 7 days, to promote cell differentiation as described previously (Noel et al., 2017), medium was replaced by a 1:1 mixture, referred hereon as HIOGM-diff, of HIOGM component A and advanced DMEM/F12 supplemented with 100 U-mg/mL penicillin-streptomycin, 7.5 mM HEPES and 0.5x Glutamax, which was refreshed every 3-4 days.

### Transepithelial Electrical Resistance Measurement

Culture medium of the 2D organoid culture was refreshed 30 min before measuring the trans-epithelial electrical resistance (TEER) with a Millicell ERS-2 voltohmmeter (Millipore), according to manufacturer's instruction. Each insert was measured three times (once in each pore) and average values were corrected for background TEER and surface area of the insert to obtain the net-area resistance in Ω^*^cm^2^.

### Permeability Assay

To evaluate paracellular permeability of the monolayers, translocation of fluorescein isothiocyanate-conjugated dextran (4 kDa, FD4, Sigma Aldrich) from apical to basolateral side was measured. Cultures were washed with Hanks' Balanced Salt Solution without phenol-red (HBSS, Lonza) and FD4 in HBSS (1 mg/ml) was added to the apical side. The amount of FD4 in both apical and basolateral chambers was determined after 4 h (unless stated otherwise) on a fluorescence multi-well plate reader (CLARIOstar or Synergy H1) with a FD4 standard curve run in parallel. Permeability is expressed as FD4 permeation rate: FD4 basolateral_t = 4h_ (μg)/FD4 apical_t = 0h_ (μg).

### Enzyme Activity Assay

Activity of alkaline phosphatase (EC 3.1.3.1) was determined by spectrophotometry with a diethanolamine assay measuring pNPP hydrolysis according to manufacturer's instruction (phosphatase substrate, ThermoFisher Scientific) and as previous described (Noda et al., 2017). Enzyme activity values are expressed in units, with one unit equals 1 μg pNPP hydrolysed/min.

### Immunohistochemistry

Transwell monolayer culture inserts were fixed in 4% formaldehyde for 1 h, embedded in HistoGel (Thermo Scientific), embedded in paraffin and sectioned. Alkaline phosphatase (ALP) activity at the brush border was identified with NBT/BCIP conversion (Sigma-Aldrich) for 30 min. After counterstaining with nuclear fast red (Sigma-Aldrich), slides were dehydrated and mounted. To visualize the mucus layer, slides were stained with Alcian Blue. Images were acquired using Olympus BX51 microscope and processed with ImageJ (version 1.52a, National Institutes of Health).

### Immunofluorescence

A list of all antibodies used can be found in Supplementary Table 1. Human fetal tissue was fixed overnight in 4% formaldehyde, embedded in paraffin and sectioned. For immunofluorescent staining, slides were deparaffinised with xylene and gradually rehydrated in ethanol. After antigen retrieval in sodium citrate buffer or Tris-EDTA buffer (20 min at 100°C) and blocking with 1% bovine serum albumin 0.1% triton-X-100 in PBS, slides were incubated overnight at 4°C with primary antibody in blocking buffer. Staining was visualized with Alexa-conjugated secondary antibody (1:500, 1 h at RT) after which the tissue slides were mounted with ProLong™ Gold antifade reagent with DAPI (Invitrogen). Images were obtained on a Leica CTR 6000 and processed with ImageJ (version 1.52a, National Institutes of Health).

Monolayer cultures were washed with PBS and fixed in 4% formaldehyde for 30 min at RT, then washed once with PBS and stored at 4°C in PBS until stained. Membranes were excised from inserts and washed twice, then permeabilized with 0.1% TRITON-X. Cells were blocked in PBS containing 10% BSA for 2 h at RT or overnight at 4°C. Cells were washed once with PBS and incubated overnight at 4°C with diluted primary antibodies in PBS containing 3% BSA. After washing three times with PBS, cells were incubated with Alexa-conjugated secondary antibody with or without 1:1,000 Phalloidin CruzFluor™ 488 Conjugate in PBS containing 3% BSA 1 h at RT. Cells were washed three times with PBS, then mounted in ProLong Diamond Antifade Mountant with DAPI (Invitrogen). Slides were stored at 4°C until imaged on a Leica TCSS SP8 X mounted on a Leica DMI6000 and analyzed using LAS X, Huygens Professional software and ImageJ (version 1.52a, National Institutes of Health).

### Scanning Electron Microscopy

Monolayer cultures were fixed for 1 h at RT in a 1:1 mixture of HIOGM-diff and 0.1 M phosphate buffer containing 4% formaldehyde and 1% glutaraldehyde, followed by overnight fixation in pure fixative. Fixed monolayers were dehydrated using an ethanol gradient from 50 to 100% and a final hexamethyldisilazane incubation. Hexamethyldisilazane was removed and monolayers were air-dried overnight, then coated with platinum/palladium using a Leica EM ACE600. Monolayers were imaged using a Zeiss Sigma-300 FE scanning electron microscope.

### RNA Isolation and RT-qPCR

Two cell culture inserts were pooled to obtain sufficient amounts of RNA and stored at −20°C in lysis buffer until isolation. RNA was isolated using the Bioline ISOLATE II RNA Mini kit (BIO-52073, Bioline) according to manufacturers' protocol. RNA (0.5 μg) was transcribed using Revertaid reverse transcriptase (Fermentas, Vilnius, Lithuania). Quantitative RT-qPCR was performed on a BioRad iCycler using sensifast SYBR No-ROX Kit (GC-biotech Bio-98020) according to manufacturer's instructions. From a panel of eleven genes, most stable reference genes were identified by GeNorm (Vandesompele et al., 2002) (Supplementary Figure 2). Relative expression levels with N0 values were obtained by LinRegPCR (Ruijter et al., 2009) and normalized to reference genes. Primer sequences are listed in Supplementary Table 2.

### Viral Cultures

Infectious virus titers of Enterovirus A71 (EV-A71) C1 91-480 were kindly provided by the National Institute for Public Health and the Environment (Bilthoven, the Netherlands). EV-A71 was cultured on RD99 cells maintained in Eagle's minimum essential medium (EMEM; Lonza) supplemented with 8% heat-inactivated fetal bovine serum (HI-FBS; Sigma-Aldrich), 100 U-mg/mL penicillin- streptomycin (Lonzo Bio Whittaker), non-essential amino acids (NEAA; ScienCell Research Laboratories) and 200 nM L-glutamine (Lonza). The 50% tissue culture infective dose (TCID50) of virus stocks and samples was determined using the Reed and Muench (1938).

Viral RNA was isolated from apical and basolateral medium samples using the Bioline Isolate II RNA mini kit according to manufacturer's protocol. Viral RNA was eluted in 60 μl elution buffer and reverse transcribed using the SuperCript II kit according to manufatcurer's instructions. Five microliters of cDNA was used for qPCR and performed on a LightCycler 480 using the SYBR Green I Master kit (Roche Diagnostics).

### Viral Infections

Apical HIOGM-diff was replaced with HIOGM-diff containing 10^3.6^ plaque-forming-units (PFU) of EV-A71. After 2 h incubation, unbound virus was removed by washing with PBS and fresh HIOGM-diff was added. At 0, 8, 24, 48, 72, and 144 h post infection a 100 μL sample was taken from the apical and basolateral compartment, sampled volume was replaced with fresh HIOGM-diff.

### Bacterial Cultures

The Dutch clinical *Listeria monocytogenes* isolate 2141805 isolated from a patient with meningitis, with serovar 1/2a and multi-locus sequence type 7, was kindly provided by Dr. A. van der Ende of the Netherlands Reference Laboratory for Bacterial Meningitis (Amsterdam UMC, Amsterdam, the Netherlands). *L. monocytogenes* was grown overnight at 37°C in TSB with 0.6% yeast extract and plated on blood agar plates. CFU was determined by 10-fold serial dilutions in PBS and plating 5x 10 μL droplets on blood agar plates.

### Bacterial Translocation Assay

HIOGM-diff was replaced by phenol-red free DMEM/F12 containing 10% HI-FBS after washing twice with PBS to remove antibiotics. After 30 min, TEER was measured to confirm monolayer integrity and 150 μL of *L. monocytogenes* was added apically at a multiplicity of infection (MOI) of 50 in phenol-red free DMEM/F12 with 10% HI-FBS and 1 mg/mL FD4. Basolateral, 800 μL phenol-red free DMEM/F12 with 10% HI-FBS was added. At t = 0 h, FD4 concentration and (CFU) was determined in 10 μL inoculum and 100 μL basolateral medium. At 2 and 5 h, 100 μL samples were taken from basolateral compartment to quantify CFU, FD4 concentration and cytokine concentrations. Additionally, at *t* = 2 h, basolateral medium was refreshed to prevent bacterial overgrowth. After 5 h, inserts were washed three times with PBS to remove unbound *L. monocytogenes* and cells were fixed in 4% formaldehyde for immunofluorescence staining as mentioned earlier. Unstimulated cultures were used as controls for each experiment.

### Cytokines

To measure inflammatory responses of the organoid monolayers, cytokine levels in the apical and basolateral medium were quantified using the BD™ Cytometric Bead Array (CBA, BD Biosciences) for human inflammatory cytokines, according to manufacturer's instructions. Data was analyzed using FLowJo software.

### Statistical Analysis

Organoids derived from five different donors were used for all experiments to obtain five biological replicates. All experiments were performed in duplicate for each donor and mean ± SEM of biological replicates was plotted, unless stated otherwise, as indicated in Figure legends. Consistently throughout all Figures, each donor has its own unique symbol. Differences between proximal and distal tissue/culture and the effect of differentiation or infection were tested with a ratio-paired *t*-test, with *p* < 0.05 considered statistically significant.

## Results

### Establishment of Human Proximal and Distal Intestinal Epithelial Monolayer Cultures

To determine which seeding concentration of cells yields the most stable monolayer, inserts with pore size 3.0 μm (insert size 0.33 cm^2^) were seeded with 0.5·10^5^, 1·10^5^, and 2·10^5^ cells harvested from proximal and distal human fetal intestinal 3D organoids. Fetal organoid cultures were cultured in the complete growth medium that was subsequently switched to differentiation medium at day 7 (see material and methods) and followed in time. Specifically, the integrity of human fetal organoid-derived epithelial monolayers in culture was evaluated by performing longitudinal measurements of trans-epithelial electrical resistance (TEER) for 28 days, a widely accepted quantitative technique to measure the integrity of tight junctions in cell culture models of epithelial monolayers (Srinivasan et al., 2015). Although seeding concertation of 0.5·10^5^ displayed slightly increased TEER in proximal cultures the optimal standardized seeding concentration that yielded the most stable TEER in both proximal (Supplementary Figure 1A) and distal (Supplementary Figure 1B) organoid-derived monolayer cultures for all five donors was 1·10^5^ cells per insert. This seeding concentration is similar to previously published protocols for insert size of 0.4 μm (VanDussen et al., 2015; Kozuka et al., 2017). In addition, based on the observed stabilization of TEER and smallest biological variation for both proximal and distal cultures, day 14 of culture was identified as time point with the optimal epithelial integrity for monolayers derived from fetal intestinal organoids.

In addition to TEER, epithelial barrier function was evaluated by measuring paracellular permeability with fluorescein isothiocyanate-conjugated dextran (4 kDa; FD4) permeation from the apical to basolateral compartment. In concordance with TEER values, FD4 permeation rate at day 7 and day 14 was lowest in cultures seeded with 1·10^5^ cells per insert for both proximal and distal monolayers (Supplementary Figures 1C–F). There was a significant negative correlation between TEER values and FD4 permeation rate for day 7 (Spearman r −0,9412, *p* < 0.0001) and day 14 (*r* −0.6763, *p* < 0,0001) (Supplementary Figures 1G,H), confirming that both TEER and FD4 can be used to assess monolayer integrity in this model.

To confirm that proximal and distal cultures represent the regional tissue differences, we analyzed gene expression profiles of known proximal and distal gut markers of intestinal tissue (Tsai et al., 2017), in the intestinal tissue of organoid origin and the epithelial monolayer cultures. To ensure accurate gene expression measurements, the stability of 11 reference genes was first tested using the GeNorm algorithm (Vandesompele et al., 2002), which identified *CYCLO* and *RPL4* as best suited reference genes for tissue (Supplementary Figure 2A) and *GAPDH* and *H2AFZ* for monolayer cultures (Supplementary Figure 2B). Relative expression levels of proximal gut markers (*ONECUT2, PDX1, AKR1B10, DMBT1, TM4SF4*) were increased in both proximal intestinal tissue (Supplementary Figure 2C) and proximal monolayer cultures (Supplementary Figure 2E). Similarly, distal markers (*GUCA2A, FABP6, MUC2, DPP4, XPNPEP2*) were highly expressed in distal intestinal tissue (Supplementary Figure 2D) and distal monolayer cultures (Supplementary Figure 2F). These findings confirm that the proximal and distal identity of organoid-derived monolayer cultures represent the tissue of origin.

Altogether, seeding 1·10^5^ cells derived from human fetal intestinal organoids per insert resulted in a stable monolayer that exhibits a tight barrier function after 14 days of culture with preservation of proximal and distal small intestinal identity.

### Induced Differentiation Improves the Barrier and Brush Border Function

**Figure 1:**
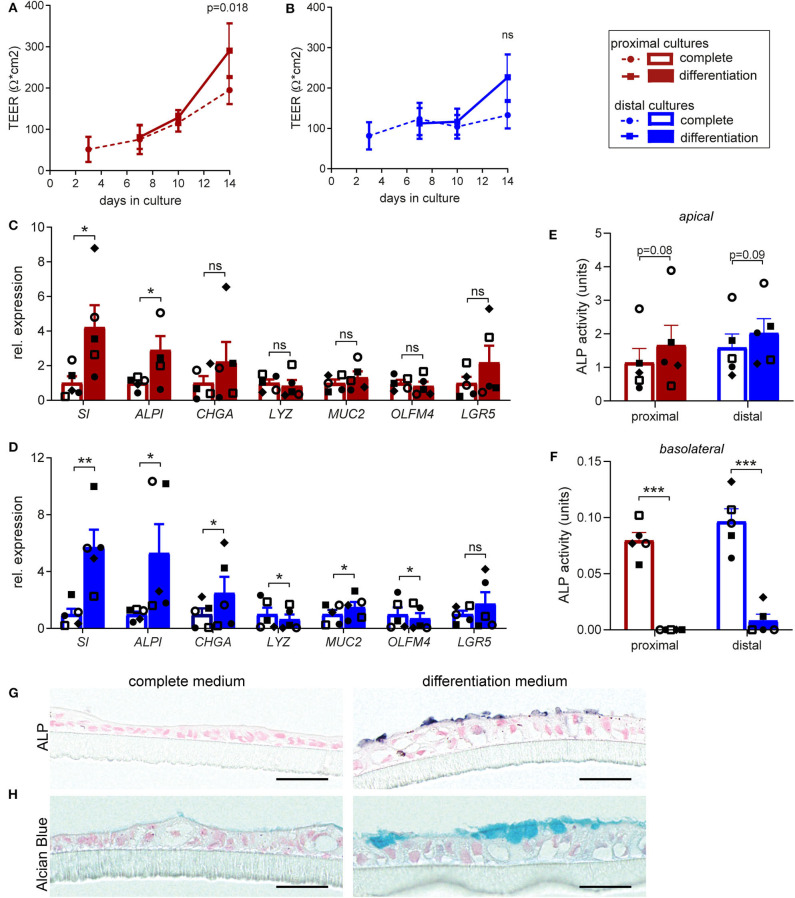
Human fetal organoid monolayer cultures display functional brush border upon differentiation. **(A,B)** TEER values for **(A)** proximal and **(B)** distal organoid monolayer cultures maintained in complete medium or from day 7 in differentiation medium. **(C,D)** Relative expression levels of epithelial cell markers in differentiated **(C)** proximal and **(D)** distal cultures, compared to the level of expression in undifferentiated cultures at day 14. **(E,F)** Alkaline phosphatase activity levels (units = μg pNPP/min) in culture supernatant of undifferentiated and differentiated proximal and distal cultures at day 14 detected in **(E)** apical medium and **(F)** basolateral medium. **(G)** Alkaline Phosphatase and **(H)** Alcian blue staining of embedded proximal transwell cultures in complete or differentiation medium at day 14 of culture. *n* = 5, values are mean ± SEM. ^*^p < 0.05, ^**^p < 0.01, ^***^p < 0.001 as determined by ratio-paired *t*-test between complete and differentiated cultures. Black scale bar equals 20 μm.

In our protocol we induce differentiation from day 7 till day 14 (see materials & methods). In order to determine in more detail whether and to which extent supplementation of full growth medium with differentiation medium improves cellular and functional differentiation of the fetal intestinal epithelial monolayer, we compared cell tropism and brush border functionality of differentiated monolayers to monolayer cultures that were maintained in complete medium for 14 days. Differentiation medium induced a slight increase in TEER for both proximal (Figure 1A) and distal monolayer cultures (Figure 1B). On a cellular level, inducing differentiation had the most robust effect on expression levels of sucrase-isomaltase (*SI*) and intestinal alkaline phosphatase (*ALPI*) which increased in proximal and distal monolayer cultures (Figures 1C,D). These two enzymes are present at the enterocyte brush border and increased expression is associated with brush border maturation (Sambuy et al., 2005). Expression of markers for enteroendocrine (*CHGA*) and goblet cells (*MUC2*) significantly increased in the distal cultures upon differentiation, with a similar trend in the proximal cultures. The expression of Paneth cell marker (*LYZ)* remained similar in the proximal monolayer culture and decreased in the distal monolayer (Figures 1C,D). *OLFM4*, a marker for stem cells and transit-amplifying cells decreased in the distal cultures upon differentiation, while the expression level of stem cell marker *LGR5* remained unchanged in both proximal and distal cultures (Figures 1C,D).

To further confirm the increase in expression of enterocyte brush border markers at functional level, enzyme activity of alkaline phosphatase (ALP) was evaluated. Differentiation induced consistent, although not statistically significant, increase in the ALP activity detected at the apical side, in both proximal (Figure 1E) and distal monolayers (Figure 1F). Interestingly, we detected minor ALP activity in the basolateral medium of non-differentiated cultures, which was lost upon differentiation. Consistently, immunohistochemical analysis (IHC) for ALP showed increased expression of protein specifically on brush border upon monolayer differentiation (Figure 1G). Importantly, alcian blue staining for mucins revealed formation of mucus layer upon monolayer differentiation (Figure 1H).

Taken together, these results indicate improved epithelial barrier formation and integrity upon differentiation, resulting in enhanced enterocyte brush border function of the fetal intestinal monolayer cultures.

### Human Fetal Organoid Derived Epithelial Monolayer Contains All Cell Types Present *in vivo*

One of the main advantages of organoids over cell lines is a cell type composition that is more comparable to the human *in vivo* setting. To elucidate whether fetal derived epithelial monolayers reflect intestinal tissue cellularity, we first evaluated the presence of the specialized intestinal cell types in fetal tissue by immunofluorescence (IF) staining (Supplementary Figure 3). In 18–20 weeks human fetal intestinal tissue goblet cells (MUC2), Paneth cells (LYZ), enteroendocrine cells (SYP), and stem cells (SOX9) were detected in both proximal and distal epithelium.

**Figure 2:**
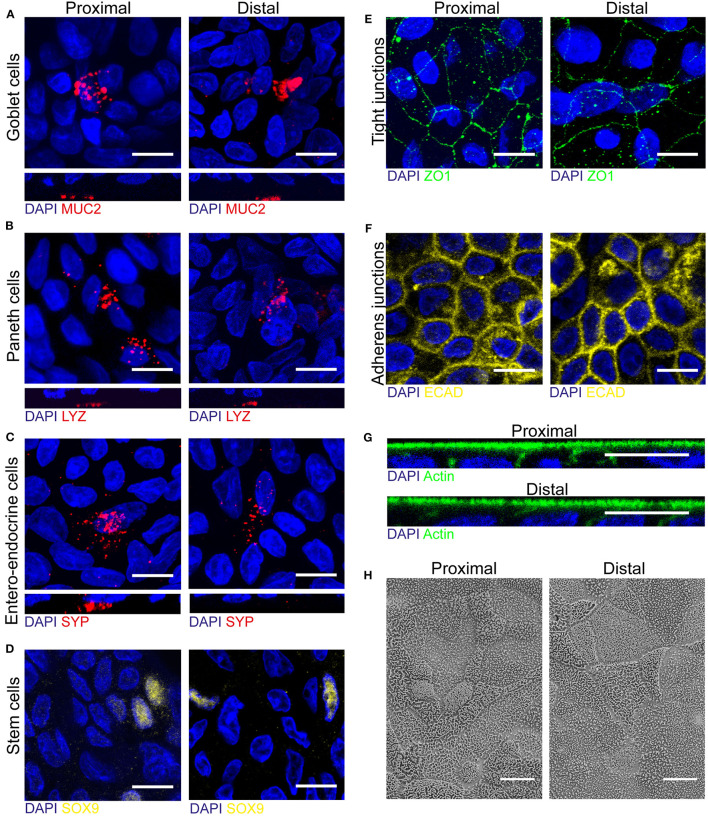
Human fetal organoid monolayer culture is polarized and contains specialized cell types and junctional complexes. **(A–D)** Proximal and distal small intestinal epithelial monolayers were stained by immunofluorescence on day 14 of culture to identify goblet cells (MUC2, red), Paneth cells (LYZ, red), enteroendocrine cells (SYP, red) and stem cells (SOX9, yellow). **(E–G)** Immunofluorescence staining to identify tight-junctions (ZO1, green), adherens junctions (ECAD, yellow) and actin (green) in both proximal and distal cultures. **(H)** Scanning electron microscopy (SEM) of apical surface of proximal and distal small intestinal epithelial monolayers. White scale bar equals 10 μm.

Next, these specialized cell types were identified in proximal and distal monolayer cultures. In addition to the identified enterocytes displaying differentiated brush border (see Figure 1), we detected MUC2 expressing goblet cells, LYZ expressing Paneth cells, SYP expressing enteroendocrine cells and SOX9 expressing stem cells in both proximal and distal monolayers (Figures 2A–D). Additionally, junctional complexes expressed at the cell-cell border such as tight junctions and adherens junctions displayed Zonula occludens-1 (ZO1) and e-cadherin (ECAD) in both proximal and distal monolayers (Figures 2E,F). Moreover, the monolayer cultures showed an apical-basolateral polarization indicated by the presence of microvilli at the apical side, which was visualized by an IF staining for actin (Figure 2G) and scanning electron microscopy (SEM) (Figure 2H). In conclusion, the intestinal monolayer cultures contained all cell types normally present within *in vivo* intestinal epithelium.

### Proximal and Distal Intestinal Monolayer Cultures Facilitate Enterovirus A71 Viral Replication

**Figure 3:**
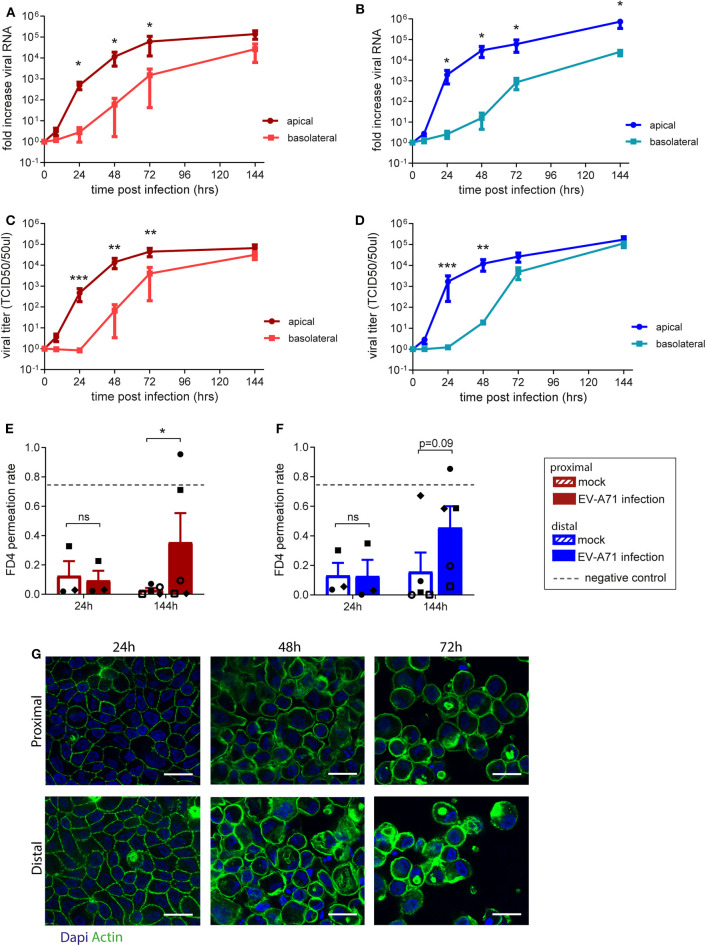
Replication kinetics of EV-A71 in human small intestinal epithelial monolayer. **(A,B)** At 0 to 144 h post infection, fold increase in viral RNA of EV-A71 in **(A)** proximal and **(B)** distal cultures was determined at regular intervals. **(C,D)** Fold increase in viral titers in **(C)** proximal and **(D)** distal cultures, at 0–144 h post infection, with titers expressed as the 50% tissue culture infective dose (TCID50)/50 μL. **(E,F)** Permeability was evaluated by permeation of FD4 from the apical to the basal compartment at 24 and 144 h post infection in **(E)** proximal and **(F)** distal cultures, with empty transwells included as negative control (indicated by dotted lines). **(G)** Visualization of the monolayer integrity by IF staining for actin (green) on both proximal and distal cultures at 24, 48, and 72 h post-infection. *n* = 5 for all except FD4 values at 24 h post-infection, where *n* = 3. Values are mean ± SEM. ^*^p < 0.05, ^**^p < 0.01, ^***^p < 0.001 as determined by paired *t*-test between apical and basolateral samples for viral replication **(A–D)** and between mock and EV-A71 infected transwell cultures **(E,F)**.

The use of fetal organoid-derived intestinal monolayer to study viral infection has been recently described (Good et al., 2019). To demonstrate that the monolayer presented here is also suitable for studying a viral gastrointestinal pathogen, infection of the monolayer with Enterovirus A71 (EV-A71) was tested. Proximal and distal epithelial monolayers were apically exposed to EV-A71 at day 14 of culture, at a multiplicity of infection (MOI) of 0.04, and viral replication over time was assessed (Supplementary Figures 4A,B). There was no viral RNA detected in the medium 8 h post-infection in both proximal and distal monolayer cultures (Figures 3A,B). After 24 h, viral RNA was exclusively detected in the apical medium (i.e., luminal side). In the basolateral medium, viral RNA was detected only after 48 h post-infection in both proximal and distal monolayers. To evaluate if shed virus was infectious, the 50% tissue culture infective dose (TCID50) at the different time points was also determined. Infectious virus titers followed a similar pattern, reaching 10^5^ infectious units 144 h post-infection in proximal (Figure 3C) and distal (Figure 3D) cultures. The integrity of the monolayer at these time points (24, 48, and 72 h) was studied by IF staining for actin in both proximal and distal cultures (Figure 3G). The monolayer was intact at 24 h but the cell layer was not contiguous in the 48 and 72 h time points. Furthermore, in a FD4 permeation assay, monolayer barrier function was intact at 24 h and disrupted by 144 h post virus inoculation as indicated by the higher FD4 permeation rate compared to mock infected monolayers, reaching similar permeation rate as empty cell culture inserts (Figures 3E,F). In conclusion, the organoid-derived epithelial monolayer presented here can be used to study infection by enteric viruses of the intestinal epithelium.

### *Listeria monocytogenes* Can Invade and Translocate Across Proximal and Distal Small Intestinal Epithelial Monolayer Cultures

**Figure 4:**
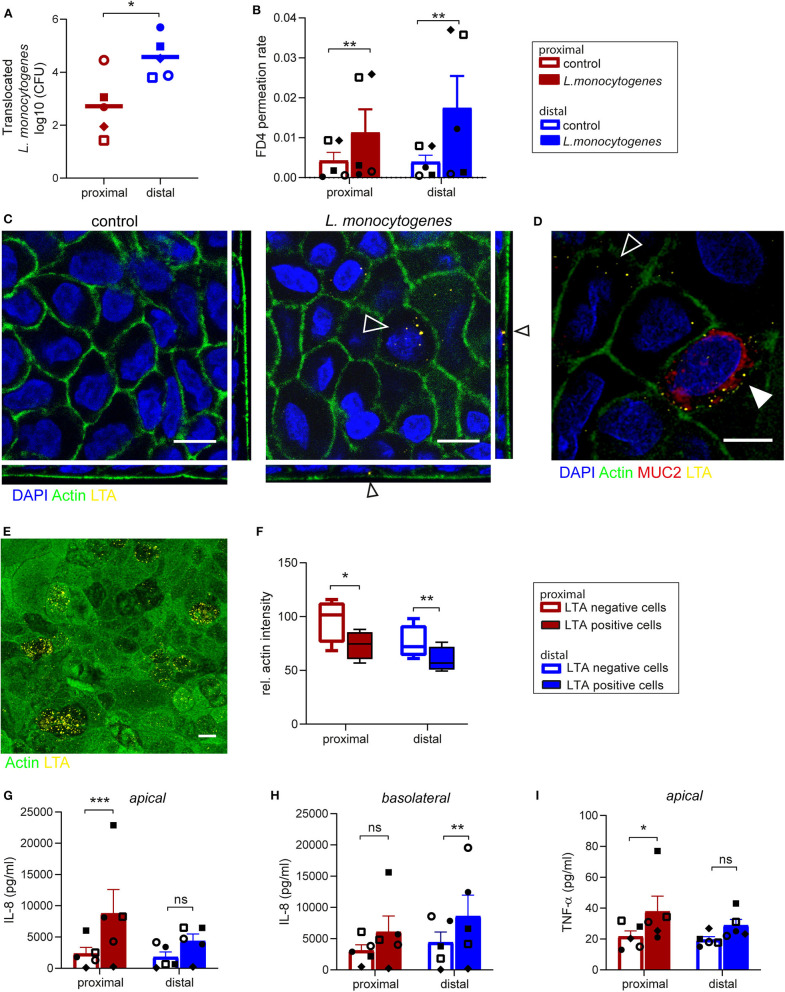
*Listeria monocytogenes* can invade and translocate across proximal and distal small intestinal epithelium. **(A)** *Listeria monocytogenes* translocation across proximal and distal small intestine epithelium 5 h post-infection determined by CFU detected in basolateral medium. **(B)** FD4 permeation rate from apical to basolateral compartment in control and infected monolayer cultures at 5 h post-infection. **(C)** Representative image of *L. monocytogenes* infected (open arrow) and uninfected small intestine epithelium after 5 h with actin (green) and LTA (yellow). **(D)** Co-localization of *L. monocytogenes* (LTA, yellow) and goblet cell marker MUC2 (red, closed arrow). **(E)** A representative image of actin redistribution in infected epithelial cells compared to uninfected cells with actin (green) and LTA (yellow). **(F)** Relative actin intensity of infected cells (ranging from 12 to 32 per image) and uninfected cells in the same maximum projection was quantified by ImageJ in a total of 4 separate images for both proximal and distal epithelium in one donor. **(G–I)** Pro-inflammatory cytokine response of **(G,H)** IL-8 and (I) TNF-α in monolayer culture supernatant 5 h post-infection, determined by CBA assay in **(G,I)** apical and **(H)** basolateral culture medium. n=5, values are mean ± SEM. ^*^p < 0.05, ^**^p < 0.01, ^***^p < 0.001 as determined by ratio-paired *t*-test. White scale bar equals 10 μm.

*L. monocytogenes* is a food-borne pathogen causing infections in humans, as it is able to cross the intestinal barrier (Drolia et al., 2018; Radoshevich and Cossart, 2018). We studied the interaction of *L. monocytogenes* with the primary intestinal monolayer culture. The ability of *L. monocytogenes* to translocate and the subsequent effect on monolayer permeability were simultaneously assessed by adding *L. monocytogenes* (MOI 50) and FD4 at the apical side at day 14 of culture (Supplementary Figures 4A,C). Five hours post-infection, *L. monocytogenes* translocated across the small intestine epithelial monolayers, with an increased translocation across the distal monolayer cultures compared to the proximal monolayer (Figure 4A). The paracellular permeability of proximal and distal epithelial monolayers was affected upon infection, with a significant increase in FD4 permeation rate in *L. monocytogenes* exposed monolayers compared to control monolayers suggesting that translocation could be in part due to the disruption of barrier function (Figure 4B). Baseline permeability was similar for both proximal and distal cultures.

Using IF staining of lipoteichoic acid (LTA), a major bacterial cell wall component, *L. monocytogenes* was found intracellularly in epithelial cells in both proximal and distal monolayers (Figure 4C). As previously reported, cells infected with *L. monocytogenes* were MUC2 positive goblet cells (Linden et al., 2008; Nikitas et al., 2011; Moorefield et al., 2018), but *L. monocytogenes* was also identified in MUC2 negative epithelial cells (Figure 4D). The fluorescence intensity of actin at the apical side appeared to be lower in epithelial cells infected by multiple bacteria compared to surrounding uninfected cells (Figure 4E and Supplementary Figure 5). Quantification showed that there was indeed a significant decrease in actin signal in infected cells compared to uninfected cells within the same monolayer culture (Figure 4F).

Next, the pro-inflammatory response to *L. monocytogenes* infection by the epithelial cells was quantified in the apical and basolateral medium. An increase in IL-8 secretion was observed in both proximal (Figure 4G) and distal cultures (Figure 4H) upon infection compared to control cultures, which was statistically significant for proximal cultures in the apical medium and distal cultures in the basolateral medium. Of note IL-1β, IL-6, IL-10, and IL-12p70 were not detected in the culture medium. In addition, *L. monocytogenes* exposure elicited an increase in TNF-α secretion at the apical side in the proximal and distal monolayer cultures compared to uninfected monolayer cultures (Figure 4I), while TNFα levels in the basolateral medium were below detection limit.

In summary, the ability of *L. monocytogenes* to invade epithelial cells of the organoid-derived monolayer model, translocate through the epithelial barrier and affect actin distribution was demonstrated, with a subsequent epithelial inflammatory response, which makes this model appealing and relevant for bacterial pathogenicity and host-microbe interaction studies.

## Discussion

This study describes the development of a stable and functional human intestinal epithelial model for enteric infections, taking the variation of biological response to pathogen into account. We demonstrated that this monolayer model preserves major characteristics of intestinal epithelium *in vivo*, including formation of a tight epithelial barrier, epithelial polarization, presence of specialized intestinal cells, and gene expression profiles matching proximal and distal fetal intestinal tissue of origin. This model was further evaluated for studying host-pathogen interactions by demonstrating the replication of the enteric virus EV-A71 and translocation of the food-borne pathogen *L. monocytogenes*.

The expanding research of host-pathogen interactions in human primary cell-derived *in vitro* models imposes the need for standardized protocols for organoid culture and analysis, to facilitate reproducibility of research findings and enable comparison of results. Here, reproducibility was evaluated taking into account biological variation in response to pathogen exposure from five different tissue specimens. Such validation approach is mandatory in host-pathogen interaction studies, considering the differences in susceptibility to infections between individuals and the differences in host-responses to a pathogen (Rajan et al., 2018).

Optimization of the seeding concentration by means of testing three different cell concentrations identified an amount of 1·10^5^ cells/0.33 cm^2^ as the most suitable density for producing a stable monolayer for both proximal and distal intestinal cultures. This seeding concentration is in line with previously found optimal concentrations (VanDussen et al., 2015; Kozuka et al., 2017). Importantly, TEER of the proximal monolayer cultures remained stable after reaching a plateau at day 10 until day 28, while the TEER in distal cultures declined after day 21 reflecting distinct properties of proximal and distal intestinal segments. Gene expression profiles confirmed that the region-specific characteristics of the fetal intestine are maintained *in vitro* which demonstrates the importance of studying the proximal and distal monolayers separately.

Epithelial monolayer cultures were infected with virus EV-A71 from the apical side, which was shown by Good et al. to be the preferred route of infection (Good et al., 2019). Over time, increasing amounts of infectious virus were detected on both apical and basolateral sides. After 72 h, viral titers reached a plateau and the effect of viral replication on epithelium integrity was evaluated. Virus was detected in the apical side after 24 h but only found in the basolateral compartment after 48 h. Actin staining of the monolayer at the later time points (48 and 72 h) revealed that the monolayer integrity is disrupted, implying that the barrier function of monolayer was lost. After 144 h, the loss of barrier function was also confirmed by the increased FD4 permeation rate. In consideration with the qPCR and TCID50 data, this indicates that virus is found on the basolateral side due to passive leakage after barrier disruption. Previous research on EV-A71 infection in an epithelial monolayer grown on membranes with 0.4 μm pore sizes did not observe disruption of epithelial barrier at day 4 post infection judged by TEER and absence of virus in the basolateral medium (Good et al., 2019), which could be attributed to the effect of the pore size on epithelial monolayer integrity or due to differences between strains. For both proximal and distal monolayers similar viral replication kinetics were found, suggesting that EV-A71 does not display a region-specific replication efficiency in the fetal small intestine.

To cause infection, enteric pathogens first need to attach to, then cross or disrupt the intestinal barrier. Data presented here showed an increased intestinal barrier permeability, as indicated by increased FD4 permeation rate, after bacterial infection with *L. monocytogenes*. This increased permeability could facilitate the paracellular translocation of *L. monocytogenes* as previously described (Kim and Bhunia, 2013). In line with this finding, a previous study showed that exposure of Caco-2 cells to *L. monocytogenes* increased the FD4 permeation rate, via remodeling of proteins involved in tight junctions and adherens junctions (Drolia et al., 2018). In addition to the paracellular route, bacteria can also translocate transcellular and the invasion of non-phagocytic cells by *L. monocytogenes* has been studied extensively (Radoshevich and Cossart, 2018). In the epithelial monolayer model presented here, *L. monocytogenes* was found intracellularly, confirming its invasive capacities. We noted the presence of multiple bacteria intracellularly in a single cell which can be caused by multiple invading events or by intracellular replication of the bacteria, as shown previously in Caco-2 cells (Kanki et al., 2018). Upon cell invasion, *L. monocytogenes* can alter host cell actin distribution (Kuhbacher et al., 2015; David and Cossart, 2017; Radoshevich and Cossart, 2018), which was observed in our study by a lower abundance of actin in the brush border of infected cells. Additionally, a remarkable difference in bacterial translocation between proximal and distal monolayer cultures was observed, with a higher microbial translocation through distal epithelium. In the current model, co-localization of *L. monocytogenes* with goblet cells was observed. E-cadherin mediated translocation via mucus expelling goblet cells has been proposed as one of the *L. monocytogenes* translocation mechanisms (Nikitas et al., 2011; Drolia and Bhunia, 2019), thus a higher abundance of goblet cells in the distal epithelium cultures could potentially explain the higher microbial translocation in distal cultures. These findings further underline the importance of studying the proximal and distal epithelium separately.

The organoids used to generate the epithelial monolayers described here were generated from primary human fetal intestinal tissue, thus representing an immature intestinal epithelium (Senger et al., 2018). Evaluation of the epithelial response after *L. monocytogenes* infection revealed a pro-inflammatory cytokine response, similar to LPS stimulation of fetal-derived organoids underlining the capability of these cultures to respond to bacterial stimuli (Claud et al., 2004; Senger et al., 2018), hence enabling studies of host-pathogen interactions in the context of impaired intestinal development. Such studies could improve our understanding of higher susceptibility toward enteric infections observed in infants compared to adults (Hornef and Fulde, 2014; Chin et al., 2017; Harbeson et al., 2018). For example in premature, malnourished and low-birth weight infants, maturation of intestinal barrier might be compromised, hence predisposing these infants to stressed gut conditions such as necrotizing enterocolitis and infectious diarrhea, respectively (Sherman, 2010; Huang et al., 2013). On the other hand, colonization with commensal microorganisms after birth is required to stimulate intestinal barrier competency to fight off external stimuli (Walker, 2002; Renz et al., 2011; Tourneur and Chassin, 2013). Unraveling the interactions between commensals vs. pathogens and the immature intestinal epithelial barrier can contribute to the prevention of the development of these conditions.

In conclusion, we describe the successful development and application of a 2D human fetal-derived intestinal organoid model. To the best of our knowledge, we are the first to describe a human small intestine 2D organoid model that can be used to study microbial translocation.

Further application of this model can revolutionize the technologies applied for compound screening related to dietary and microbial metabolites as well as other enteric pathogens and commensal gut bacteria. Additionally, the 2D epithelial monolayer can be an adequate system to investigate the transport and permeability across intestinal barrier as well as cross talk between intestinal monolayer and mesenchyme and immune cells. Furthermore, with respect to animal ethics in research, this model can help to reduce animal use in medical and nutritional research and be a valuable tool for studying human enteric pathogens for which a suitable animal model is not available yet.

## Data Availability Statement

All data are included in the article and Supplementary Material and are fully available without restriction.

## Ethics Statement

Tissues were obtained with approval of the ethical committee of the Amsterdam UMC, together with approval of the experimental procedures by the HIS Mouse Facility (Amsterdam UMC). All methods were performed in accordance with the relevant guidelines and regulations, as stated in the Amsterdam UMC Research Code, in a certified laboratory (ISO15189 accreditation M304). The patients/participants provided their written informed consent to participate in this study.

## Author Contributions

TR, MN, IR, RE, DP, KW, CS, KA, AS, and VM: conception and study design. TR, MN, and IA: data acquisition and analysis. TR, MN, KA, AS, and VM: interpretation of the data. TR, MN, and VM: drafting the work. IA, IR, RE, DP, KW, CS, KA, and AS: critical revision of the manuscript. All authors have approved the submitted version.

## Conflict of Interest

IA is employee of Viroclinics Xplore, IR is employee of Danone Nutricia Research. The remaining authors declare that the research was conducted in the absence of any commercial or financial relationships that could be construed as a potential conflict of interest.
