# Supplementary material for: A Human 2D Primary Organoid-Derived Epithelial Monolayer Model to Study Host-Pathogen Interaction in the Small Intestine
Source: Front Cell Infect Microbiol. 2020 Jun 9;10:272. doi: 10.3389/fcimb.2020.00272 (PMC7326037; doi:10.3389/fcimb.2020.00272)
Supplement: Supplementary file 1 [file Data_Sheet_1.PDF]

Supplementary Figure 1

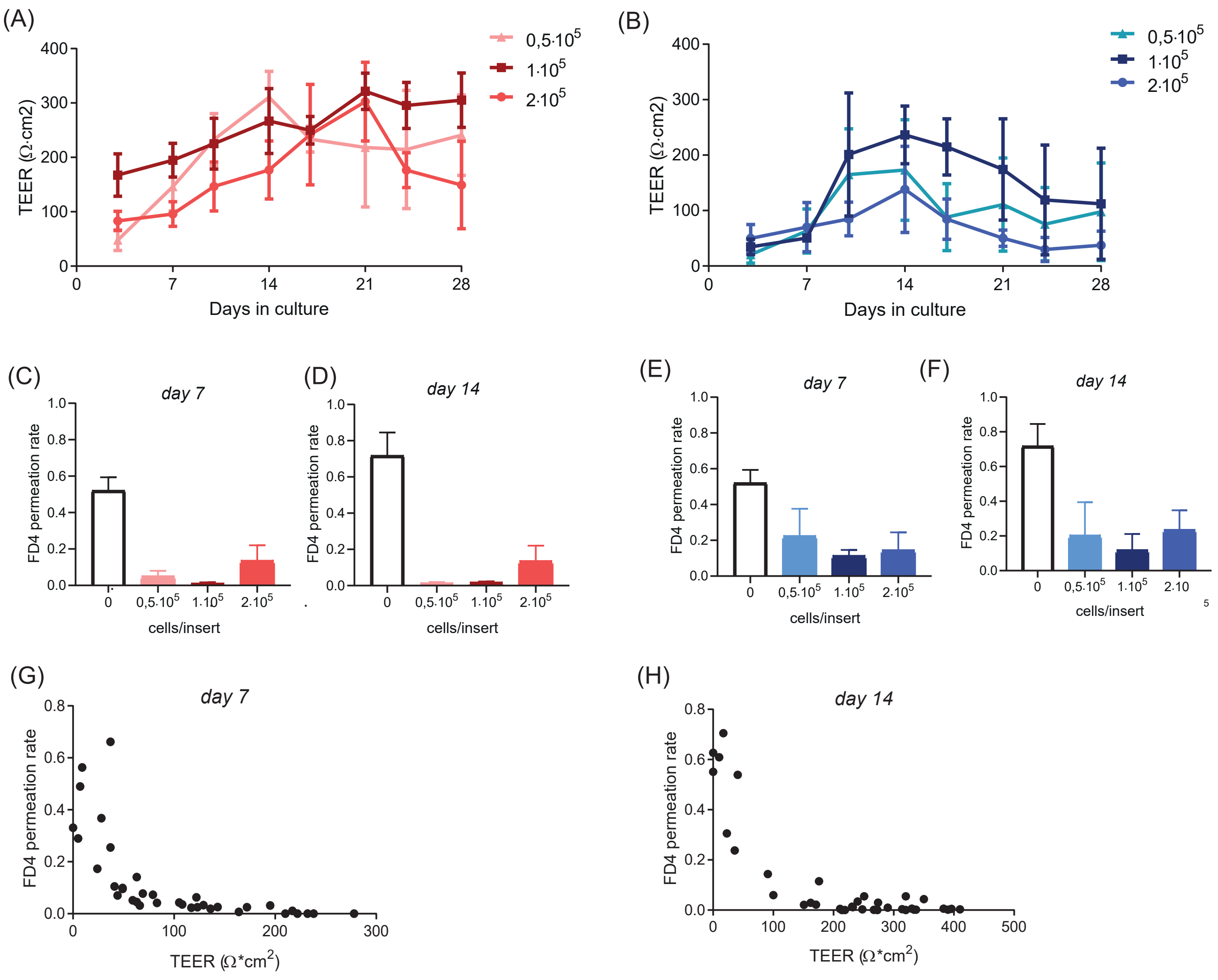

Supplementary Figure 1: Human fetal organoids generate a functional epithelial monolayer in 14 days.

(A-B) Trans epithelial electrical resistance (TEER) for different seeding concentrations was measured over time in (A) proximal and (B) distal cultures. (C-F) Permeability was determined by FD4 permeation rate at day 7 and day 14 to confirm the formation of an intact epithelial monolayer. (G-H) Spearman correlation plot of TEER and FD4 values. n=3, values are mean ± SEM.

Supplementary Figure 2

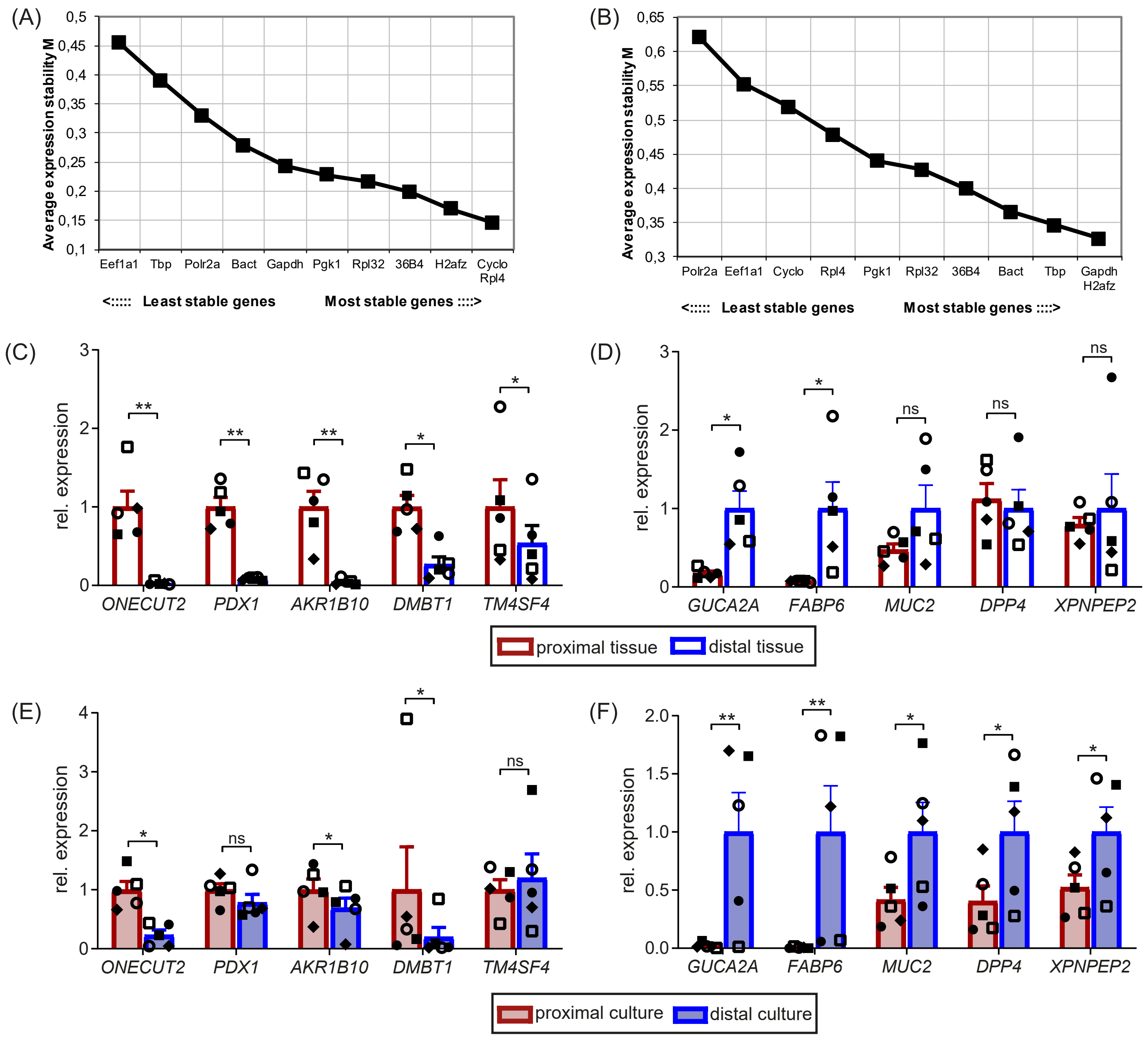

Supplementary Figure 2: Reference gene stability and relative expression of regional specific markers in fetal small intestinal tissue and fetal organoid monolayer cultures. Expression stability values (M) of reference genes, determined by GeNorm, for (A) fetal intestinal tissue and (B) fetal intestinal organoid monolayer cultures, and relative expression levels of proximal and distal markers in fetal tissue samples (C and D) and fetal organoid monolayer at day 14 of culture (E and F) determined by RT-qPCR. n=5, values are mean ±SEM. *p<0.05, **p<0.01 as determined by ratio-paired t-test between proximal and distal tissue or cultures.

Supplementary Figure 3

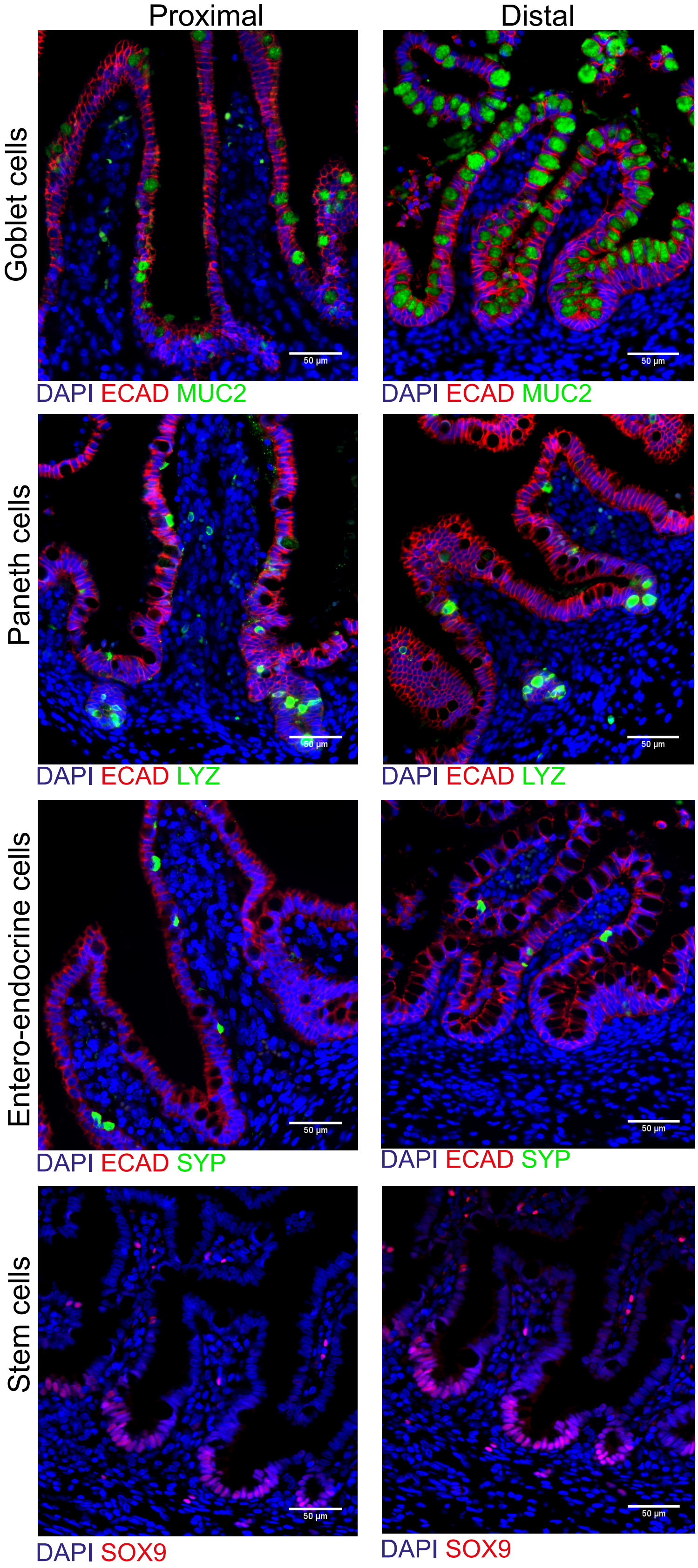

Supplementary Figure 3: Epithelial cell types identified in human fetal intestinal tissue. Representative images of specific epithelial cell types identified in human fetal proximal and distal tissue (gestational age 18-20 weeks). Immunofluorescence detection of goblet cells (MUC2), Paneth cells (LYZ), enteroendocrine cells (SYP) and, stem cells (SOX9).. Representative images of n=3 biological replicates. White scale bar equals 50 µm.

Supplementary Figure 4

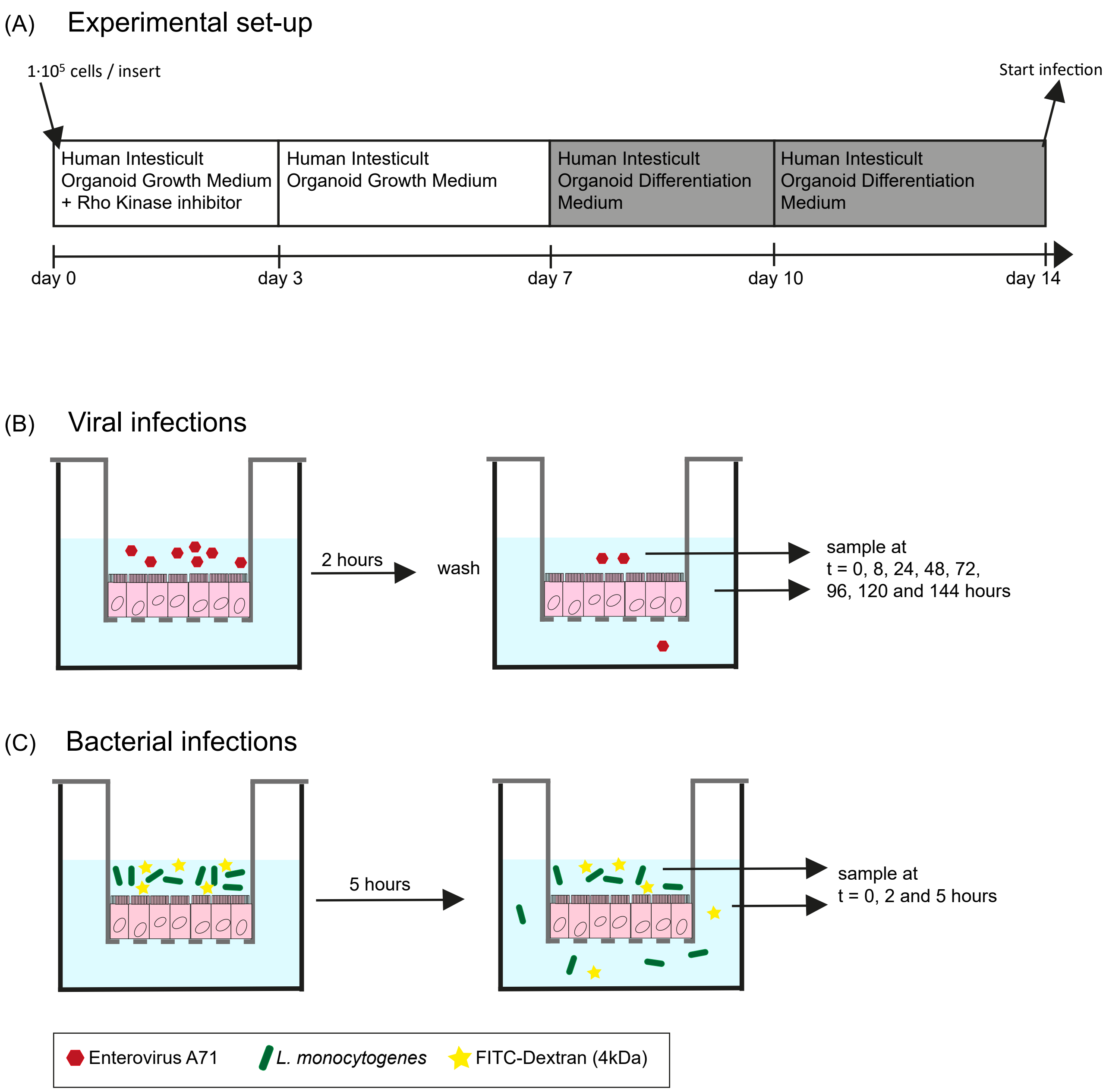

Supplementary Figure 4: Experimental set-up organoid monolayer culture and host-pathogen experiments (A) Schematic representation of optimal culture scheme, (B) viral infection protocol and (C) bacterial translocation protocol.

Supplementary Figure 5

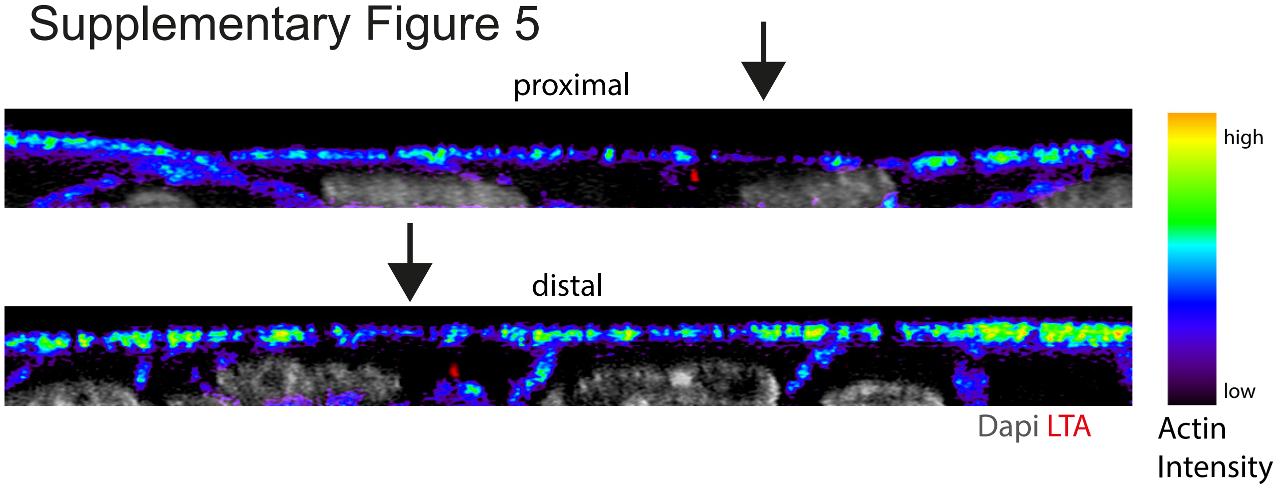

Supplementary Figure 5: Apical actin is decreased in *L. monocytogenes* invaded cells. IF staining of distal and proximal intestine epithelium after 5h of *L. monocytogenes* (MOI50) with nuclei (grey), LTA (red) and actin (pseudocolours). Related to Fig 4E. Black arrows indicate decreased apical actin in *L. monocytogenes* invaded cells.

Supplementary Table 1: List of antibodies used

anti	Host	Product.nr.	Company	Antigen retrieval	dilution	label
Primary antibodies						
E-cadherin	Mouse	610181	BD transduction	NaCi	1:200	
LTA	Mouse	15711	QED Bioscience	-	1:500	
Lysozyme	Rabbit	PA5-16668	Thermo Fisher	NaCi	1:500	
Muc2	Rabbit	Sc-15334	Santa Cruz	NaCi	1:500	
Synaptophysin	Rabbit	A0010	DAKO	Tris-EDTA	1:200	
Sox9	Rabbit	AB5535	Millipore	Tris-EDTA	1:10000	
Sox9	Mouse	14-9765-82	Thermo Fisher	-	1:100	
Sucrase-isomaltase	Goat	Sc-27603	Santa Cruz	NaCi	1:250	
Villin	Goat	Sc-7672	Santa Cruz	NaCi	1:100	
ZO1	Rat	R40.76	Santa Cruz	-	1:100	
Secondary antibodies and probes						
Rabbit IgG	Goat	A11008	Invitrogen		1:500	AlexaFluor 488
Rabbit IgG	Goat	A-21070	Thermo Fisher		1:500	AlexaFluor 633
Rabbit IgG			DAKO		1:200	Biotin
Rat IgG	Goat	A-11006	Thermo Fisher		1:500	AlexaFluor 488
Mouse IgG	Goat				1:500	AlexaFluor 647
Mouse IgG	Goat	A-21424	Thermo Fisher		1:500	AlexaFluor 555
Goat IgG	donkey	A21447	Invitrogen		1:500	AlexaFluor 647
streptavidin		F0422	DAKO		1:500	FITC
Phalloidin	-	sc-363791	Santa Cruz		1:1000	CruzFluor 488

Supplementary Table 2: List of primers used in RT-qPCR

Gene	Forward primer (5’-3’)	Reverse primer (5’-3’)	NM number
Reference genes			
36B4	TCATCAACGGTACAAACGA	GCCTTGACCTTTTCAGCAAG	NM_001002; NM_053275
βACT	AGAGCTACGAGCTGCCTGAC	AGCACTGTGTTGGCGTACAG	NM_001101
CYCLO	CACCGTGTTCTTCGACATTG	TTCTGCTGTCTTTGGGACCT	NM_001300981; NM_021130
EEF1A1	ACATCCACACACTGTTGAAGGA	ATGTTGCCTGATGCCTGGATA	NM_004280
GAPDH	AAGGTGAAGGTCGGAGTCAA	AATGAAGGGGTCATTGATGG	NM_001289746; NM_001289745; NM_002046
H2AFZ	CCTCACCGCAGAGGTACTTG	GTTGCAAGTGACGAGGGGTA	NM_002106
PGK1	CCACTGTGGCTTCTGGCATA	ATGAGAGCTTTGGTTCCCCG	NM_000291
POLR2A	CGGAGATTGTCACCCCCTTC	CCATCACACATGTGCCGTTC	NM_000937
RPL32	TGACAACAGGGTTCGTAGAAG	GCGGTTCTTGGAGGAAACATTG	NM_000994; NM_001007073; NM_001007074
RPL4	TGGCCAGGGTGCTTTTGGAA	AGCAAAACAGCTTCCTTGGTCT	NM_000968
TBP	TCTCATGTACCCTTGCCTCC	GTGCACAAATAATGCCCCTT	NM_003194; NM_001172085
Regional markers			
AKR1B10	ATCACCGTTACGGCCTACAG	CGTGCTGGTGTCACAGACTT	NM_020299
DMBT1	AGCACCAACCTGCTCTGTCT	GTCATTGTCTGCCTGCTTGA	NM_004406.2
DPP4	TCCCGGTGGGAGTACTATGA	CAGGGCTTTGGAGATCTGAG	NM_001935.4
FABP6	GGCAAGTTCGAGATGGAGAG	TTGCTTTCCTTGCCAACAGT	NM_001040442; NM_001445; NM_001130958
GUCA2A	GTAGCAACCCGAACTTTCCA	TGCAGGAGAAAAGAGCTTCC	NM_033553
MUC2	TGTAGGCATCGCTCTTCTCA	GAGTCCATCCTGCTGACCAT	NM_002457
ONECUT2	ACCGGCATAGAACCCTCTGT	GGCAGCTGGAAGAGATCAAC	NM_004852
PDX1	CGTCCGCTTGTTCTCCTC	CCTTTCCCATGGATGAAGTC	NM_000209
TM4SF4	GGAAGCGGTGTCTTGATGAT	TGAGGCATTTAGGACCCTTG	NM_004617.3
XPNPEP2	CCTGGTATGGGGATCAGAGA	ACTGGCTCGAAGGTTGAAGA	NM_003399.6
Epithelial celltypes			
ALPI	TCAGCTGGGTACTCAGGGTC	ATCGCCACTCAGCTCATCTC	NM_001631
CHGA	TGACCTCAACGATGCATTTC	CTGTCCTGGCTCTTCTGCTC	NM_001275; NM_001301690

LGR5	AATCCCCTGCCCAGTCTC	CCCTTGGGAATGTATGTCAGA	NM_003667; NM_001277226; NM_001277227
LYZ	GCTGGAGACAGAAGCACTGA	GTGGATCACGGACAACCCTC	NM_000239.2
MUC2	TGTAGGCATCGCTCTTCTCA	GAGTCCATCCTGCTGACCAT	NM_002457
OLFM4	CTCCATGATGTCAATTCGGA	CAGAGTGGAACGCTTGGAAT	NM_006418
SI	GGATGGTCACAGATGAAACCT	TCCTCCCCATCGTCCACTA	NM_001041
Virus			
EV-A71	GGCCCTGAATGCGGCTAAT	GGGATTGTCACCATAAGCAGCC	HA 11350962; HA 11350963
